# Altered expression of LINC03091 and LINC03090 LncRNAs in bipolar disorder: a case-control study

**DOI:** 10.1038/s41598-025-26426-x

**Published:** 2025-11-26

**Authors:** Zeynab Ganji Zeitooni, Zeinab Shirvani-Farsani, Bahar Naghavi Gargari

**Affiliations:** 1 Department of Cell and Molecular Biology, Faculty of Life Sciences and Biotechnology, Shahid Beheshti University, Tehran, IR Iran; 2 Department of Genetics, School of Medicine, Shahid Beheshti University of Medical Sciences, Tehran, IR Iran; 3 Department of Basic Sciences, School of Nursing and Midwifery, Shahid Beheshti University of Medical Sciences, Tehran, IR Iran

**Keywords:** Bipolar disorder, LncRNA, LRRC2-AS1, LINC03090, LINC03091, Gene expression, Biomarker, Biomarkers, Diseases, Genetics, Neuroscience

## Abstract

Long non-coding RNAs (lncRNAs) are widely expressed and play an essential role in gene regulation through various transcriptional and post-transcriptional mechanisms. Recent findings have highlighted the role of lncRNAs in sustaining cellular homeostasis and neurogenesis within the brain. An increasing number of reports have identified dysregulated lncRNAs linked to psychiatric disorders, including bipolar disorder (BD). We analyzed the expression levels of LRRC2-AS1, LINC03091, and LINC03090 lncRNAs in the blood samples of 50 patients with BD and 50 healthy individuals matched by age, sex, and ethnicity. RNA extraction and cDNA synthesis were performed, followed by real-time polymerase chain reaction to quantify lncRNA expression levels. Receiver operating characteristic (ROC) curve analysis was used to assess the biomarker potential. Furthermore, the relationship between gene expression levels and BD comorbidities was explored. Our findings revealed a significant enhancement in LINC03091 and LINC03090 expression in patients with BD compared with healthy subjects (*P* < 0.0001 and *P* = 0.02, respectively). However, the expression levels of LRRC2-AS1 was not significant (*P* = 0.69). ROC curve analysis indicated that LINC03091 (AUC = 0.74, *P* < 0.0001) and LINC03090 (AUC = 0.64, *P* = 0.01) expression levels could effectively differentiate patients from healthy controls. Considering these results, LINC03091 and LINC03090 may have a crucial role in BD and could serve as biomarkers for diagnostic and predictive applications.

## Introduction

Bipolar disorder (BD) is a group of psychiatric disorders that mainly manifest with hypomania or mania and episodes of depression^1^. BD usually develops in early adulthood affects approximately 2.4% of the world’s population^2^ during the most productive period. Patients with BD also have higher rates of suicide^3^, other psychiatric illnesses, and associated medical complications such as osteoporosis, diabetes, metabolic syndromes, and cardiovascular and endocrine disorders^4,5^.

Various studies have implicated genetic factors in the onset of BD^6,7^. Twin studies have shown a heritability of approximately 70% in patients with BD^8^, indicating the critical role of genetic factors in this disorder. Long non-coding RNAs (lncRNAs) are among the genetic factors that have recently gained recognition. LncRNAs are a class of RNA transcripts longer than 200 nucleotides that have limited or no protein-coding potential. They play roles as critical epigenetic, transcriptional, and post-transcriptional regulators of gene expression. In the brain, lncRNAs are recognized for their functions in neuronal development, neuronal differentiation, synaptic plasticity, and regulation of neuroinflammatory pathways, key processes implicated in the pathophysiology of mood disorders and psychosis^9–11^. The first study on the expression profile of lncRNAs in the nerve tissue of patients with BD was conducted in 2014. During that time, the LINC00173 lncRNA was identified as the first lncRNA that showed increased expression in patients with BD^12^. In another postmortem study on the tissue of three brain regions, including the orbitofrontal cortex (BA11), anterior cingulate cortex (BA24), and dorsolateral frontal cortex (BA9), of patients with bipolar disorder and schizophrenia in 2016, 20 types of lncRNA were differentially expressed in patients with bipolar disorder. In patients with BD, some of these lncRNAs are expressed with proteins related to functions such as synaptic transmission, exploratory motor behavior, and innate immune response activation^13^. Although early studies were performed on postmortem neural tissues, in recent years, peripheral blood has become an available source for the discovery of biomarkers that provide valuable information about pathogenic processes in the central nervous system^14^. Other studies have discovered abnormal expression levels of certain lncRNAs, specifically lincRNA-p21, MEG3, GAS5, and FOXD3-AS1^15–17^, in the bloodstream of patients with BD, indicating their potential influence on BD pathobiology. To identify additional lncRNAs associated with BD, we conducted a literature review and selected LRRC2-AS1, LINC03091, and LINC03090 lncRNAs for comparison of their expression levels between BD cases and control subjects. Although these lncRNAs are linked to BD-associated pathways, their roles in BD have yet to be investigated. For example, LRRC2-AS1 is an antisense lncRNA with 633 nucleotides. The LRRC2-AS1 promoter is located in the first intron of the LRRC2 gene and is transcribed from a part of the LRRC2 gene. LRRC2 protein is a member of synaptic adhesion molecules containing leucine-rich repeats, which are critical for synapse formation, function, and specificity^18^. Given that synaptic dysfunction is a critical pathophysiological feature of BD, dysregulation of LRRC2-AS1 could potentially disrupt LRRC2 expression or function and thus contribute to the synaptic deficits observed in this disorder^19,20^. This hypothesis is supported by the observation of damage and mutations in this region in patients with schizophrenia^21^, a disorder with shared genetic and pathological features with BD. Furthermore, the dysregulation of LRRC2-AS1 is associated with Alzheimer’s disease and autism spectrum disorder (ASD), introducing it as a potential transdiagnostic element in neuropsychiatry^22,23^. Similarly, both LINC03091 and LINC03090 have been identified in studies of autism spectrum disorder ASD and show differential expression compared to typically developing individuals^23^. The high degree of genetic and phenotypic overlap between ASD and bipolar disorder, particularly in areas such as emotion regulation and social cognition^24^, suggests that molecular mechanisms may also be shared. The involvement of these lncRNAs in a neurodevelopmental disorder such as ASD suggests a potential role in brain development and circuit formation, processes that are also implicated in the early origins of bipolar disorder. Therefore, examining LINC03091 and LINC03090 in bipolar disorder allows us to examine whether their dysregulation represents a common pathway across different psychiatric conditions or is specific to specific diagnostic boundaries.

In light of the available associative data and the absence of an evaluation regarding their role in BD, we examined the expression profiles of LRRC2-AS1, LINC03091, and LINC03090 in the peripheral blood of patients with this condition. This study represents the first investigation of the expression of these three lncRNAs in an Iranian population and aims to assess their potential as biomarkers while contributing to the understanding of lncRNA-mediated mechanisms in BD.

## Materials and methods

### Study participants

This case-control study included 50 individuals diagnosed with bipolar disorder according to DSM-5 criteria^19^ and 50 healthy controls matched by age and sex. Peripheral blood samples were collected from all participants at the Behavioral Center of Imam Hosein Hospital after obtaining informed consent. The control group consisted of volunteers with no psychiatric illness history. Both groups excluded participants with neurological conditions like epilepsy, seizures, head trauma, meningitis, cancer, and current substance abuse. The patient group had an average age of 36.5 years (70% male, 30% female), while the healthy controls had an average age of 34.08 years (70% male, 30% female). The average disease duration and age of onset for patients were 3.86 years and 32.64 years, respectively.

The study protocol received approval from the Ethical Committee of Shahid Beheshti University of Medical Sciences (IR-SBMU.PHNM.1395.667).

## RNA isolation and cDNA synthesis

Peripheral blood mononuclear cells (PBMCs) were isolated from 5 mL of EDTA-treated venous blood using a Ficoll gradient solution (Tcell, Talaye TebAzma, Iran). Total RNA was extracted from the PBMCs using the RNX kit (EX6101, Cinnagen, Iran). The purity and concentration of RNA were analyzed spectrophotometrically (Nanodrop, Thermo Scientific) by measuring absorbance at 260/280 nm. To remove genomic DNA contamination, the RNA samples were treated with DNase I (Yekta Tajhiz Azma, Iran). We then performed cDNA synthesis with three µg of purified total RNA using a High-Capacity cDNA Reverse Transcription Kit (Applied Biosystems, PN: 4375575) in a 20 µL reaction volume, following the manufacturer’s instructions.

## Quantitative Real-Time PCR (qRT-PCR)

We designed gene-specific primers using Gene Runner and Oligo7 software. Beta-2 microglobulin (B2M) was used as a reference gene for normalization and as a negative control. We carried out quantitative PCR on a StepOne Plus detection system (Applied Biosystems, USA) using SYBR Green chemistry. We calculated the relative expression levels of the target genes using the comparative ΔΔCt method, with results expressed as fold change (2^–ΔΔCt)^20^. The sequences of all primers used are listed in Table 1.

**Table 1:** Primers used in real time PCR.

Gene name	ACCESSION	Primer sequence	Primer length	Product length (bp)
**LRRC2-AS1**	**NR_073385**	F: TCA GAT AGC AGC TCA GGA GT R: GTA GCG TTC CTT TAT GGA GAC	20 21	89
**LINC03091**	**NR_199718**	F: GGA TGA AAG GAA AGA CAG AGG R: AAC AAC AAA AAC CCC ACA ACG	21 21	140
**LINC03090**	**XR_007079283**	F: ATC CTG AGT CAC GGC CAA AC R: TAA CGG AGA GGT CAG GGC TT	20 20	150
**B2M**	**NM_004048**	F: AGA TGA GTA TGC CTG CCG TG R: CGG CAT CTT CAA ACC TCC A	20 19	104

### Statistical analysis

Data analysis was performed using GraphPad Prism 8 (GraphPad Software). The Kolmogorov-Smirnov test confirmed data normality. Gene expression (LRRC2-AS1, LINC0309, and LINC03090) was compared between patients and controls using an unpaired t-test or Mann-Whitney test, as appropriate. Correlations between gene expression and clinical characteristics (age, disease duration, age of onset) were analyzed by Pearson’s correlation and regression. The biomarker potential (sensitivity and specificity) of the genes was evaluated via ROC curve analysis. Data are reported as mean ± standard deviation (SD); P-values < 0.05 were deemed significant. A post-hoc Bonferroni correction was applied to account for multiple comparisons across the three lncRNAs analyzed (LRRC2-AS1, LINC03091, LINC03090), setting the adjusted significance level at *P* < 0.0167.

## Results

### Gene expression

**Fig. 1:**
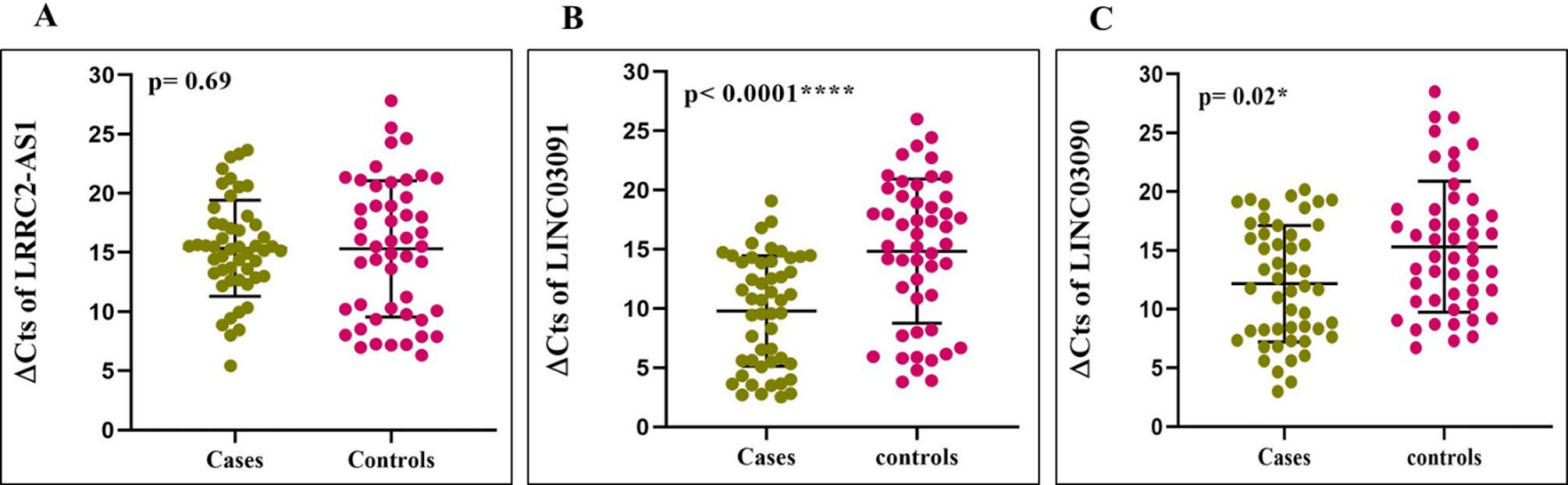
Expression analysis of lncRNAs in the PBMCs. The ∆Cts of lncRNAs LRRC2-AS1 (**A**), LINC03091 (**B**), and LINC03090 (**C**) in the blood samples of BD patients and healthy subjects. lncRNA expression levels in each sample were normalized to B2M expression. * *P* < 0.05, **** *P* < 0.0001.

The graphical representation of LRRC2-AS1, LINC03091, and LINC03090 expression levels in 50 patients with BD and 50 healthy subjects is indicated in Fig. 1A-C. The expression level of LRRC2-AS1 has decreased by 1.33 times in BD cases compared with healthy controls which it was not a significant difference *P* = 0.69). However the LINC03091 level (*P* < 0.0001, fold change = 26.61) significantly increased in patients compared to healthy subjects. Also the expression level of LINC03090 (*P* = 0.02, fold change = 6.96) in bipolar patients was significantly higher than in healthy controls. Following a post-hoc Bonferroni correction for three comparisons, the significance for LINC03091 remained robust (*P* < 0.0001), while the result for LINC03090 was attenuated (*P* = 0.06).

Furthermore, there was no significant difference in the expression level of LRRC2-AS1 between the male (*P* = 0.33) and female patients (*P* = 0.59) groups compared to the counterpart controls groups. Furthermore, there was no significant difference in the LINC03091 (*P* = 0.20) and LINC03090 (*P* = 0.95) expression level between the female patients and female controls. However, the LINC03091 (*P* < 0.0001****) and LINC03090 (*P* = 0.0043) expression level significantly increased in male patients compared to male controls (Table 2). Remarkably, the expression of LINC03091 (*P* = 0.0003) and LINC03090 (*P* = 0.0035) have been lower in female cases compared with male cases. Furthermore, the expression of LINC03091 (*P* = 0.58), LINC03090 (*P* = 0.89), andLRRC2-AS1 (*P* = 0.61) was not different between female controls and male controls as well as LRRC2-AS1 (*P* = 0.32) was not different between female cases and male cases (Table 2).

**Table 2:** Results of the expression of LncRNAs in BD patients and healthy controls.

lncRNAs		Total Cases vs. Controls (50 vs. 50)	Male Cases vs. Male Controls (35 vs. 35)	Female Cases vs. Female Controls (15 vs. 15)	Female Cases vs. Male Cases (15 vs. 35)	Female controls vs. Male Controls (15 vs. 35)
LRRC2-AS1	Mean ∆Ct	15.36 vs. 14.94	15.68 vs. 14.54	14.63 vs. 15.90	14.63 vs. 15.68	15.90 vs. 14.54
	Fold Change (2^−∆∆Ct^)	1.33	−2.20	2.42	2.06	−2.58
	Adjusted P Value	0.69	0.33	0.59	0.32	0.61
LINC03091	Mean ∆Ct	9.80 vs. 14.53	8.41 vs. 14.90	13.04 vs. 13.67	13.04 vs. 8.41	13.67 vs. 14.90
	Fold Change (2^−∆∆Ct^)	26.6	90	1.5	−24.85	2.34
	Adjusted P Value	< 0.0001****	< 0.0001****	0.20	0.0003***	0.58
LINC03090	Mean ∆Ct	12.16 vs. 14.96	10.84 vs. 15.005	15.26 vs. 14.88	15.26 vs. 10.84	14.88 vs. 15.005
	Fold Change (2^−∆∆Ct^)	6.96	17.91	−1.3	−21.40	1.08
	Adjusted P Value	0.02*	0.0043**	0.95	0.0035**	0.89

Significant values (*P* < 0.05) are highlighted in bold. A minus (-) denotes a decrease in the expression.

## Correlation analysis

The correlation between the expression levels of all gene pairs was assessed using the Pearson correlation test. Table 3 presents the findings from this analysis. No significant correlation was found between LRRC2-AS1/LINC03091 (*P* = 0.95) and LRRC2-AS1/LINC03090 (*P* = 0.91) gene expressions. However, there was significant positive correlations between LINC03091/LINC03090 (*P* < 0.0001) gene expression. Furthermore, there was no significant correlation was found between LRRC2-AS1, LINC03091, and LINC03090 in bipolar patients and age, disease duration, and age of onset (Table 3).

**Table 3:** Pairwise correlation between LncRNA expression levels in the case group and correlation between expression levels of LncRNAs and demographic data.

Variables	LRRC2-AS1		LINC03091		LINC03090	
	*r*	*P*-value	*r*	*P*-value	*r*	*P*-value
**Age**	0.04	0.77	−0.25	0.07	−0.15	0.28
**Disease duration**	0.13	0.34	−0.04	0.76	−0.16	0.23
**Age of onset**	0.002	0.98	−0.23	0.10	−0.12	0.39
**LINC03090**	−0.01	0.91	**0.75**	< 0.0001****	-	-
**LINC03091**	−0.007	0.95	-	-	-	-

## ROC curve analysis

**Fig. 2:**
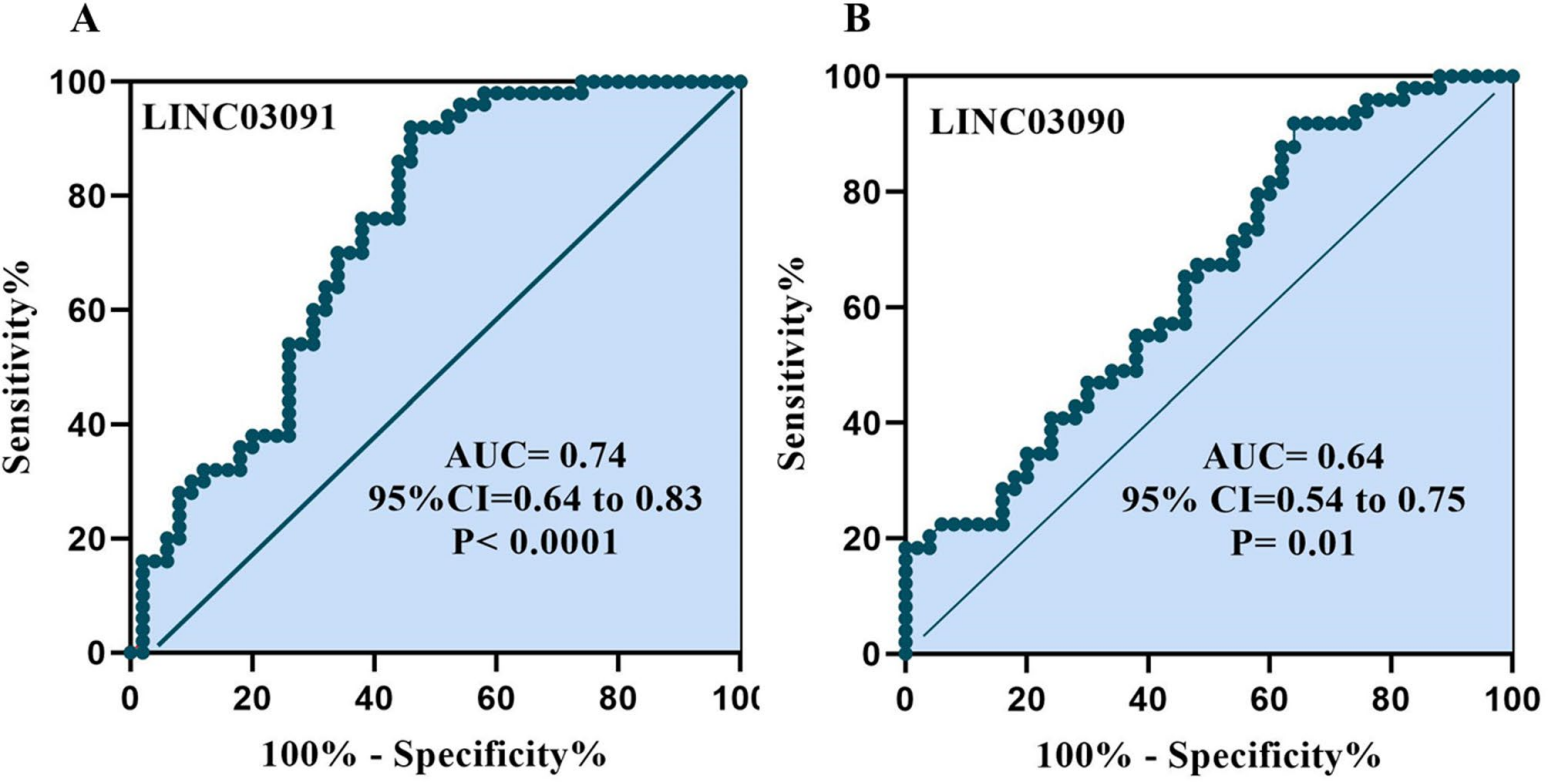
ROC curve analysis of LINC03091 (**A**) and LINC03090 (**B**). AUC: area under the curve.

The specificity and sensitivity of lncRNA LINC03091 and LINC03090 expression in 50 BD patients and 50 healthy subjects were evaluated by ROC curve analysis to show the BD diagnosis power. The areas under the ROC curve (AUC) for LINC03091 was 0.74 (*P* < 0.0001) and for LINC03090 lncRNA was 0.64 (*P* = 0.01), respectively (Fig. 2A, B). The criterion values (cutoff values) of LINC03091 and LINC03090 were 14.03 and 11.59, respectively. We found that LINC03091 with 76% sensitivity and 62% specificity and LINC03090 with 71% sensitivity and 60% specificity can be used as potential biomarkers for clinical diagnosis of BD.

## Discussion

Many studies have demonstrated the role of lncRNAs in brain development and differentiation, such as maintaining pluripotency, determining cell fate, neurogenesis, and migration^9^. Additionally, lncRNAs influence the regulation of neural genes in non-neural cells and contribute to the patterning of brain tissue samples^25^. Previous research has highlighted that lncRNA dysregulation is a significant factor in the onset of various disorders. Gene expression, a quantitative trait with a complex nature, often exhibits variability in results related to gene expression levels across different studies. This variability can be attributed to several factors, including data analysis methods, tissue-specific attributes, sample size, study design, experimental methodologies, and population diversity^26^. A thorough comprehension of these factors is essential when comparing and interpreting the findings of different research studies.

In this investigation, we have selected three lncRNAs to quantify their levels in the blood of bipolar patients and examine their potential as biomarkers. We observed a significant upregulation of LINC03090 and LINC03091 in BD individuals compared to controls. However, our findings indicated that there was no significant change in the expression patterns of LRRC2-AS1, between the patients and healthy controls. Furthermore, we detected a significant positive correlation between the expressions of LINC03091 and LINC03090, indicating a possible co-regulatory association between these lncRNAs or shared upstream regulator. While the precise molecular functions of LINC03091 and LINC03090 remain unclear, their significant disruption in BD raises speculation about their potential involvement in key disease pathways. Based on the existing literature linking other lncRNAs to BD pathophysiology, we can hypothesize several mechanisms. First, a major area of interest is synaptic plasticity. Given that synaptic dysfunction is a cornerstone of BD pathology and contributes to the observed cognitive deficits and mood instability, it is plausible that these lncRNAs may affect genes involved in synaptic transmission, neurite outgrowth, or synaptic scaling^11,27^. Future studies should investigate whether LINC03091 or LINC03090 target key synaptic genes or modulate the activity of synaptic adhesion molecules.

Second, the known role of neuroinflammation in BD provides another compelling context. It has been shown that peripheral and central inflammatory signals are altered in BD patients^28,29^. Dysregulation of these lncRNAs in the blood could reflect or contribute to this inflammatory state. For example, they may regulate the expression of proinflammatory cytokines or genes involved in the innate immune response in glial cells or peripheral immune cells^30,31^. Their potential interaction with sex hormones, as discussed below, could further modulate this inflammatory pathway, given the immunomodulatory effects of hormones such as estrogen and testosterone. Different levels of LINC03091 and LINC03090 were also indicated between males and females. Moreover, the dramatic effect of sex hormones on the expression of neuronal genes encoding neuropeptides was previously demonstrated^32^. The relationship of testosterone level with mood, behavior and risk of suicide in mental patients has been shown before. Testosterone level is also positively correlated with the number of manic episodes and suicide attempts^33,34^. In addition, the levels of lncRNAs are strongly correlated with sex hormones^35,36^, which may indicate the effect of sex hormones testosterone or the specific function of these lncRNAs in males. The level of testosterone in men with BD decreases significantly, while it increases significantly in women with BD^34^. Consequently, LINC03090 and LINC03091 may be affected by and interact with sex hormones progesterone/estrogen. However, more functional studies are needed to explore the mechanisms of interaction between lncRNAs and sex hormones.

Notably, expression of LINC03091 and LINC03090 had diagnostic values in distinguishing between BD and healthy controls. Today, the diagnostic role of lncRNAs for various diseases, including psychiatric disorders, has received much attention^37^. Owing to the great importance of faster diagnosis of BD worldwide, improved scientific knowledge about its pathobiology and molecular mechanisms may lead to valuable diagnosis and effective treatment^38^. However, it should be noted that although blood-based biomarkers are easily and clinically obtained, they are usually influenced by various factors such as medical co-morbidities and lifestyle habits (e.g. physical activity level, nicotine consumption, etc.). According to this, we do not expect the regulation of these lncRNAs in the brain to be the same as in peripheral blood, Their expression in blood can serve as an accessible, albeit indirect, indicator of systemic pathological processes associated with BD^39^. Optimal use of current genetic data is needed to help provide new insights into the pathogenesis of BD and develop more effective methods for early diagnosis and treatment. It is also noteworthy that, unlike LINC03091 and LINC03090, LRRC2-AS1 expression was not altered in our BD group. This suggests that its dysregulation may not be a feature of BD, or that its role may be more exact, specific to a BD subtype, or restricted to a different tissue. Reporting this negative result is important for the clarity and reproducibility of research in this field and helps to build a more complete picture of lncRNA involvement in psychiatric disorders.

In conclusion, our case-control study provides evidence for significant dysregulation of two specific lncRNAs, LINC03091 and LINC03090, in the peripheral blood of patients with BD. The strong association of LINC03091 with BD and its promising discriminatory power suggest its potential utility as a diagnostic biomarker and warrant further investigation. Our findings pave the way for future functional studies to definitively determine whether LINC03091 and LINC03090 are involved in BD through mechanisms of synaptic plasticity, neuroinflammation, or other major pathways.

This study has limitations that should be noted. First, the small sample size, although sufficient to detect large effect sizes, as observed in LINC03091, may limit the statistical power to detect smaller effects. The reduced significance of LINC03090 after correction for multiple testing highlights this point. Therefore, our findings, especially for LINC03090, should be interpreted with caution and require validation in larger, independent cohorts. Second, although we matched our groups for age, sex, and ethnicity and conducted additional analyses that showed no significant confounding effect of disease duration, we cannot completely rule out the influence of other unmeasured variables. The lack of standardized symptom severity scores for all participants and the lack of complete information on the type and dosage of medications used are other limitations, and future studies should systematically consider such clinical data to better understand the relationship between lncRNA expression and disease phenotype. Finally, our study is population-based and lacks functional validation. Altered expression of LINC03091 and LINC03090 in blood, although promising as a potential biomarker, does not clarify their functional role in the pathophysiology of BD. It remains unclear whether these changes are a cause or consequence of the disorder and whether they reflect similar changes in the central nervous system. Therefore, future research should evaluate the study of these lncRNAs in more samples, postmortem brain tissues or cerebrospinal fluid samples, and at the cellular level.

## Data Availability

The datasets used and/or analyzed during the current study are available from the corresponding author on reasonable request.

## Abbreviations

lncRNAs: Long non‑coding RNAs
BD: Bipolar disorder
ROC: Receiver Operating Characteristic
AUC: Area Under the Curve
ASD: autism spectrum disorder
PBMCs: Peripheral blood mononuclear cells
